# Frosted branch angiitis and cerebral venous sinus thrombosis as an initial onset of neuro-Behçet’s disease: a case report and review of the literature

**DOI:** 10.1186/s13256-017-1261-z

**Published:** 2017-04-15

**Authors:** Bruno Fortaleza de Aquino Ferreira, Ever Ernesto Caso Rodriguez, Leandro Lara do Prado, Celio Roberto Gonçalves, Carlos Eduardo Hirata, Joyce Hisae Yamamoto

**Affiliations:** 1 Department of Ophthalmology, University of Sao Paulo, Dr. Eneas Carvalho de Aguiar Avenue, Sao Paulo, 255 Brazil; 2 Department of Rheumatology, University of Sao Paulo, Dr. Eneas Carvalho de Aguiar Avenue, Sao Paulo, 255 Brazil

**Keywords:** Case report, Retinal vasculitis, Neuro-Behçet’s disease, Cranial sinus thrombosis, Uveitis

## Abstract

**Background:**

Frosted branch angiitis is a rare, severe condition. It can be either a primary or a secondary condition and is characterized by rapid deterioration of vision and fulminant retinal vasculitis that manifests as diffuse sheathing of retinal vessels, macular edema, papillitis, vitritis and anterior uveitis. We aimed to describe a case of frosted branch angiitis and cerebral venous sinus thrombosis as an initial neuro-Behçet’s disease onset. Diagnosis of Behçet’s disease was based on the current 2014 International Criteria for Behçet’s Disease and the International consensus recommendation criteria for neuro-Behçet’s disease. In addition, a literature review using search parameters of “frosted branch angiitis”, “Behçet” and “neuro-Behçet” in the PubMed database is presented.

**Case presentation:**

A 28-year-old Brazilian pardo woman presented to our hospital with abrupt bilateral vision loss associated with recurrent aphthous oral ulcers 6 months before visual symptom onset. A fundus examination showed bilateral widespread retinal vasculitis with venous and arterial white sheathing, optic disc swelling, macular edema, and retinal hemorrhages, leading to the diagnosis of frosted branch angiitis. An extensive systemic workup for retinal vasculitis was uneventful, except for brain magnetic resonance imaging demonstrating cerebral venous sinus thrombosis and lymphocytic aseptic meningitis. A diagnosis of neuro-Behçet’s disease was made, and treatment was started with methylprednisolone therapy 1 g/day for 5 consecutive days, followed by oral mycophenolate mofetil and infliximab 5 mg/kg infusion. The patient’s response was rapid, with improvement of visual acuity to hand movement and counting fingers by day 7 and final visual acuity of counting fingers and 20/130.

**Conclusions:**

Frosted branch angiitis may be associated with infectious, noninfectious, or idiopathic causes. An extensive workup should be done to exclude systemic vasculitis such as Behçet’s disease. Treatment with systemic steroids must be promptly initiated in association with specific treatment aimed at inflammation control and blindness risk reduction.

## Background

Frosted branch angiitis (FBA) is a rare, severe condition first described by Ito *et al.* [1]. It can be either a primary or a secondary condition. The disease is characterized by rapid deterioration of vision and fulminant retinal vasculitis that manifests as diffuse sheathing of retinal vessels, macular edema, papillitis, vitritis and anterior uveitis [2].

We aimed to describe a case of a patient with FBA and cerebral venous sinus thrombosis (CVST) as an initial neuro-Behçet’s disease (NBD) onset. The diagnosis of BD was based on the current 2014 International Criteria for Behçet’s Disease (ICBD) [3] and on the international consensus recommendation criteria for NBD (ICR) [4]. In addition, a literature review using search parameters of “frosted branch angiitis”, “Behçet” and “neuro-Behçet” in the PubMed database is presented.

## Case presentation

**Fig. 1:**
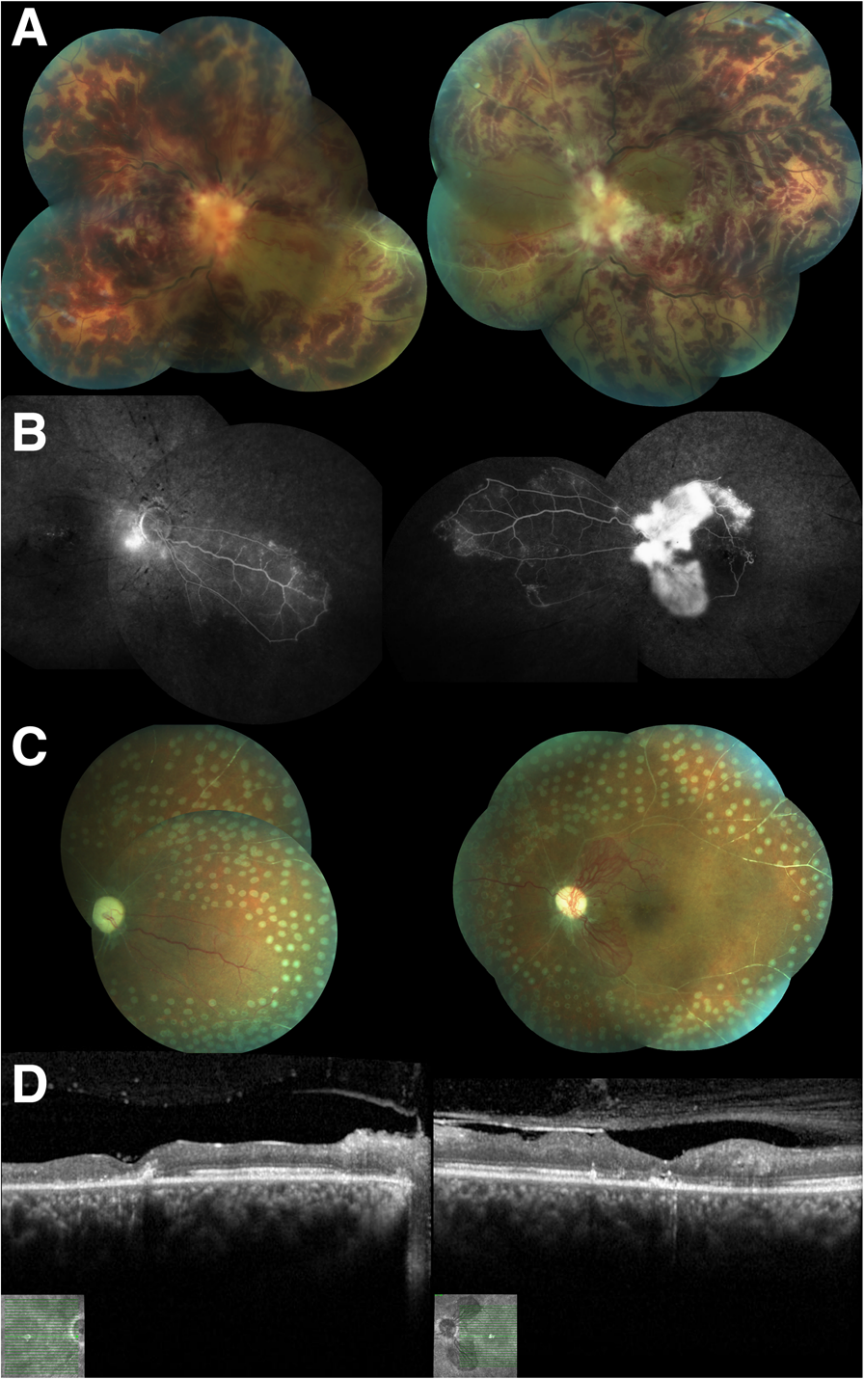
Posterior segment imaging of the case. **a** Fundus picture taken at the beginning of follow-up showing widespread retinal vasculitis with venous and arterial white sheathing, bilateral optic disc swelling, macular edema, and retinal hemorrhages, characterizing frosted branch angiitis. **b** Fluorescein angiography performed after 4 months demonstrating extensive capillary nonperfusion hypofluorescence and neovascularization leakage. **c** Panretinal photocoagulation avoiding perfused areas: in the right eye, nasal retina; in the left eye, left macula. **d** Spectral domain optical coherence tomography demonstrated a thin central retina in both eyes with fragmentation of outer retinal layers

A 28-year-old Brazilian pardo woman presented to our hospital with sudden bilateral vision loss of 1 day’s duration, which had been preceded by 6 months of recurrent aphthous oral ulcers and 2 months of daily diffuse headache. She had no other past medical history. On examination, her visual acuity (VA) was hand movement in both eyes. She had bilateral, mild, nongranulomatous iridocyclitis. A fundus examination showed bilateral widespread retinal vasculitis with venous and arterial white sheathing, optic disc swelling, macular edema, and retinal hemorrhages (Fig. 1a).

**Fig. 2:**
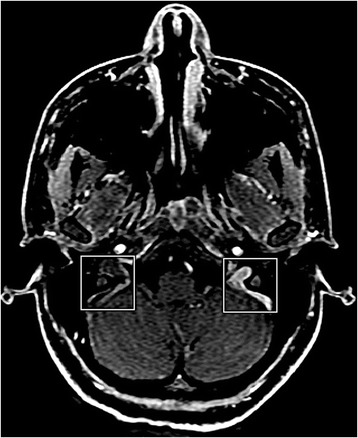
Brain magnetic resonance imaging study showing cerebral venous sinus with signs of thrombosis on right (*squares*)

An initial diagnosis of FBA was made, and an extensive systemic workup for retinal vasculitis was performed. Complete blood count, erythrocyte sedimentation rate, C-reactive protein, liver and kidney function tests, angiotensin-converting enzyme, immunoglobulins and chest x-ray results were normal. Antinuclear and antineutrophil cytoplasmic antibody screening results were negative. The test result for human leukocyte antigen (HLA)-B51 was negative. Cerebrospinal fluid analysis revealed increased cells (84% lymphocytes) and increased opening pressure. Serologies for infectious disease were uneventful, including human immunodeficiency virus, cytomegalovirus, herpes simplex virus, varicella zoster virus, Epstein-Barr virus, toxoplasmosis, syphilis, and hepatitis. The results of the patient’s tuberculin skin test was negative. Aqueous humor was analyzed for herpes simplex virus, cytomegalovirus and toxoplasmosis by polymerase chain reaction, which was unyielding. Brain magnetic resonance imaging showed thrombosis of the superior sagittal sinus, sigmoid sinus, and internal jugular vein (Fig. 2).

Within 48 hours, the patient’s VA deteriorated to light perception in both eyes. Pulse methylprednisolone therapy 1 g/day for 5 consecutive days was instituted, followed by oral prednisone (1 mg/kg/day). By day 7, her VA improved to finger counting, and her fundus widespread retinal vasculitis ameliorated. By fluorescein angiography (FA), a prolonged arteriovenous phase was observed with extensive areas of blocked (retinal hemorrhages) and perivascular/optic disc leakage. Spectral domain optical coherence tomography (SD-OCT) revealed a diffuse retinal edema in both eyes, which significantly improved after 21 days of therapy.

With rheumatology division consultation, a diagnosis of NBD with initial onset as FBA and CVST was made. Oral prednisolone was tapered until given daily at 25 mg, and associated therapy with mycophenolate mofetil (1 g daily) and infliximab (5 mg/kg/day) infusion was administered. Oral anticoagulation with warfarin was combined during the first 6 months to prevent intracranial hypertension and was discontinued after disease activity control. Weight gain secondary to systemic corticosteroids and axillary folliculitis were the main adverse events of immunosuppression. A fundus examination done a few months after the onset showed, in both eyes, pale optic discs, diffuse vessels whitening and, in the left eye, an extensive sea fan disc neovascularization. By FA, an extensive nonperfusion area in both eyes was observed (Fig. 1b). Panretinal photocoagulation was indicated in both eyes (Fig. 1c). SD-OCT demonstrated a thin central retina (153 μm in right eye, 184 μm in left eye) (Fig. 1d).

## Discussion

Until 1988, there were no FBA cases outside Japan reported in the literature. Only 57 cases had been recorded in the world’s medical literature until 2004, 75% of which were from Japan. To date, including the PubMed database, fewer than 90 cases can be found. FBA is usually a bilateral disorder and it occurs more frequently in women (61%) [2]. It has a bimodal age distribution, with a peak between 6 and 16 years (Japan) and in the third decade of life (worldwide), ranging from 2 to 42 years old [2, 5]. Several agents and disorders have been reported as causal or triggering agents of FBA, including pregnancy [5–9].

FBA can be idiopathic or associated with ocular and systemic diseases. Kleiner *et al.* classified FBA into three subgroups [10, 11]. The first group is characterized by the “frosted branch appearance” with a white vessel sheathing caused by infiltration of the vessel walls by malignant cells (acute lymphoblastic leukemia and large cell lymphoma), simulating a vasculitis. The second group is characterized by the “frosted branch response” with vasculitis secondary to cytomegalovirus and other intraocular infections (herpes simplex virus, toxoplasmosis, syphilis, human immunodeficiency virus, and *Fusarium dimerum*) or autoimmune diseases (systemic lupus erythematosus, Crohn’s disease, BD, sarcoidosis, and multiple sclerosis) [2, 5, 7]. The third group is “acute idiopathic FBA”, in which an infectious precedent (a viral illness in 33%) is responsible for triggering the vasculitis [2]. Reynders *et al.* [6] characterized primary idiopathic FBA as follows: healthy patients, infectious prodrome, acute visual loss, uveitis, vessel sheathing, FA demonstrating late staining and/or leakage and areas of capillary nonperfusion (there is no occlusion), prompt response to corticosteroids, excellent visual recovery and no recurrence. Multifactorial prodromal illness leading to hypersensitivity reaction (immune complex deposition) is the hypothesis most accepted for this subtype [2, 5].

BD is classified in primary vasculitis as a variable vessel vasculitis affecting vessels of any kind and type. The etiopathogenesis of the disease remains unknown [4]. Although disease rates and clinical expression vary to some extent by ethnic origin, recurrent mucocutaneous lesions, skin lesions, ocular findings and reactivity of the skin to needle prick or injection (pathergy test) constitute common clinical hallmarks of BD [4, 12]. Because there is a lack of a universally recognized pathognomonic test, BD diagnosis is based primarily on clinical criteria. Oral aphthosis, genital aphthosis, cutaneous lesions (such as inflammatory papulopustular lesions, erythema nodosum, and skin ulcers) and positive pathergy (PP) reaction, as well as uveitis/retinitis/hypopyon/iritis, have been used in several sets of diagnostic criteria. Interestingly, though, not only do rates of PP reaction vary widely in different populations, but also there are indications that its sensitivity is declining over time [13]. Therefore, this test is not always conducted in daily practice, in spite of the high diagnostic specificity [14].

However, to overcome these problems, the International Team for the Revision of the International Criteria for Behçet’s Disease recently proposed a new set of criteria (the ICBD criteria) based on clinical data from 2556 patients with BD and 1163 control subjects from 27 countries [3]. These criteria consider oral aphthosis, genital aphthosis and ocular lesions with each given 2 points, whereas 1 point is assigned to each of skin lesions, vascular manifestations and neurological manifestations. A patient scoring 4 points or above is classified as probable BD, 5 points as BD highly likely, and 6 or more points as almost certainly BD [3]. The ICBD criteria demonstrated an estimated sensitivity of 95%, considerably higher than that of the original International Study Group (ISG) criteria (85%), and specificity of 91% as compared with 96% for the ISG criteria.

In NBD, central nervous system (CNS) is the usual site of neurological involvement. There are two main categories of CNS involvement: parenchymal neuro-Behçet’s disease (P-NBD) and nonparenchymal neuro-Behçet’s disease (NP-NBD) [4, 15–20]. ICR for NBD established diagnostic criteria for BD with CNS involvement [4].

Thus, on the basis of ICBD criteria [3], our patient scored 6 points (oral aphthosis [2 points], ocular lesions [2 points], neurological manifestations [1 point] and vascular manifestations [1 point]) and was classified as “almost certainly BD”. In addition, according to the ICR for NBD [4], our patient was classified as “definite NBD”.

Neurological involvement is one of the most serious causes of long-term morbidity and mortality in BD [5]. The frequency of neurological involvement in BD is very variable; in hospital-based series, percentages as low as 1.3% and as high as 59% [15] have been reported, but these data are likely to be biased for various reasons (for example, study design, definition of neurological involvement, ethnic or geographic variation, availability of neurological expertise and investigations, and treatment protocols). The age of onset of NBD is usually between 20 and 40 years.

Neurological manifestations commonly develop a few years after the onset of the other systemic features of BD; the mean duration between onset of BD and development of NBD ranges from 3 to 6 years [15, 16]. However, the first systemic symptoms of BD might coincide with neurological presentation, as was demonstrated in our patient. Four studies reported that neurological presentation preceded other systemic features of BD (6% of patients) [15, 21]. Moreover, prompt treatment of neurological manifestations may inhibit development of other BD clinical features.

NP-NBD is characterized by CVST, intracranial hypertension and aneurysms. CVST constituted about 10% to 20% of NBD [15, 18, 19] characterized by thrombosis of the venous sinuses, especially superior sagittal sinus, leading to increased intracranial pressure with headache, papilledema, cranial nerve palsies and mental changes [15, 18–20]. CVST in BD usually occurs relatively slowly, but acute onset of seizures and focal neurological symptoms is sometimes seen [19]. Occurrence of CVST together with primary CNS parenchymal lesions in the same patient is rare [16, 18]. Overall, patients with NP-NBD (CVST) have a better neurological prognosis than those with P-NBD.

Eye involvement occurs in 43% to 65% of patients with BD; vitritis and retinal vasculitis are the most common manifestations. However, FBA is rare [22]. Including our patient, to the best of our knowledge, there are only ten cases of FBA in BD reported to date [5–7, 22–27]. In most of these cases, the ocular manifestation was the hint for the diagnosis of BD, as in our patient. Five case reports of NBD were presented, and two cases revealed typical hypopyon iritis. All patients had a history of recurrent oral or genital ulceration, and seven of nine cases had a positive HLA-B51 (Table 1). Our patient was HLA-B51-negative; however, its positivity ranges from 50% to 64% in BD [2].

**Table 1:** Characteristics of patients diagnosed with frosted branch angiitis as an ocular manifestation of Behçet’s disease

Authors, year [reference]	Country	Past history	Laterality	Initial BCVA (OD/OS)	Final BCVA (OD/OS)	HLA-B51	Hypopyon	Neuro-Behçet disease
Reynders *et al.*, 2005 [6]	Belgium	OrU, GU, skin lesions	OU	20/400, CF	20/40, 20/200	+	(−)	(−)
Renard *et al.*, 2009 [23]	France	OrU, skin lesions	OD	20/200	20/20	+	(−)	(−)
Jackson *et al.*, 2011 [24]	United Kingdom	OrU, GU, skin lesions	OS	HM	20/60	+	(−)	(−)
Ramachandran *et al.*, 2011 [22]	United Kingdom	OrU, GU	OU	NA	^a^	(−)	+	+
Al-Mujaini *et al.*, 2011 [7]	Oman	OrU, GU, skin lesions	OU^b^	20/100, NA	^a^	NA	(−)	+
Portero *et al.*, 2011 [5]	Spain	OrU, skin lesions	OU	20/200, 20/120	20/20, 20/20	+	(−)	+
Kwon *et al.* 2012 [26]	Korea	OrU, GU	OS	HM	HM	+	+	(−)
Kumar *et al*., 2015 [25]	India	None	OD	LP	20/60	+	(−)	(−)
Schwatz *et al.*, 2016 [27]	Israel	OrU, GU, skin lesions	OD	20/100	20/22	+	(−)	+
Present case	Brazil	OrU, headache	OU	HM, HM	CF, 20/130	(−)	(−)	+

*Abbreviations: BCVA* Best corrected visual acuity, *OrU* Oral ulcers, *GU* Genital ulcers, *OU* Both eyes, *OD* Right eye, *OS* Left eye, *CF* Counting fingers, *HM* Hand movement, *LP* light perception, *NA* Not available, *HLA* Human leukocyte antigen

^a^Described as clinical improvement

^b^Two years apart

FBA subgroups must be identified in order for proper treatment to be given [5, 10, 11]. Secondary FBA associated with autoimmune disease requires systemic steroids for its treatment [2, 8]. Conversely, treatment of NBD is based on high doses of pulsed glucocorticoids, usually three to seven pulses of intravenous methylprednisolone 1 g/day, given during attacks, followed by maintenance of oral glucocorticoids tapered over 2–3 months. Immunosuppressants (azathioprine 2.5 mg/kg/day, cyclophosphamide, interferon-α, and tumor necrosis factor blockers) may also be given to prevent recurrences and disease progression [21]. Most patients with FBA (85%) have improvement of VA. Complications may be responsible for VA of 20/200 or worse in 10%, despite glucocorticoid treatment. They include macular epiretinal membrane, retinal vessel occlusion, retinal fibrosis, retinal tears, vitreous hemorrhage and optic disc atrophy. Our patient presented with disc and iridocorneal angle neovascularization, requiring prompt panretinal photocoagulation (Fig. 1c).

## Conclusions

To the best of our knowledge, this is the first reported case of FBA and CVST as an NBD presentation. Exhaustive investigation is mandatory before considering FBA to be idiopathic, and systemic vasculitis, such as BD, should be considered in the differential diagnosis. Treatment with systemic glucocorticoids and immunosuppressants must be promptly initiated, with aims of inflammation control and blindness risk reduction.

## Abbreviations

BCVA: Best corrected visual acuity
BD: Behçet’s disease
CF: Count fingers
CNS: Central nervous system
CVST: Cerebral venous sinus thrombosis
FA: Fluorescein angiography
FBA: Frosted branch angiitis
GU: Genital ulcers
HLA: Human leukocyte antigen
HM: Hand movement
ICBD: 2014 International Criteria for Behçet’s Disease
ICR: International consensus recommendation criteria for neuro-Behçet disease
ISG: International Study Group
LP: Light perception
NA: Not available
NBD: Neuro-Behçet’s disease
NP-NBD: Nonparenchymal neuro-Behçet’s disease
OD: Right eye
OrU: Oral ulcers
OS: Left eye
OU: Both eyes
P-NBD: Parenchymal neuro-Behçet’s disease
PP: Positive pathergy
SD-OCT: Spectral domain optical coherence tomography
VA: Visual acuity
